# Chromium(VI) Toxicity in Legume Plants: Modulation Effects of Rhizobial Symbiosis

**DOI:** 10.1155/2018/8031213

**Published:** 2018-02-14

**Authors:** Uliana Ya. Stambulska, Maria M. Bayliak, Volodymyr I. Lushchak

**Affiliations:** Department of Biochemistry and Biotechnology, Vasyl Stefanyk Precarpathian National University, 57 Shevchenko Str., Ivano-Frankivsk 76018, Ukraine

## Abstract

Most legume species have the ability to establish a symbiotic relationship with soil nitrogen-fixing rhizobacteria that promote plant growth and productivity. There is an increasing evidence of reactive oxygen species (ROS) important role in formation of legume-rhizobium symbiosis and nodule functioning. Environmental pollutants such as chromium compounds can cause damage to rhizobia, legumes, and their symbiosis. In plants, toxic effects of chromium(VI) compounds are associated with the increased production of ROS and oxidative stress development as well as with inhibition of pigment synthesis and modification of virtually all cellular components. These metabolic changes result in inhibition of seed germination and seedling development as well as reduction of plant biomass and crop yield. However, if plants establish symbiosis with rhizobia, heavy metals are accumulated preferentially in nodules decreasing the toxicity of metals to the host plant. This review summarizes data on toxic effects of chromium on legume plants and legume-rhizobium symbiosis. In addition, we discussed the role of oxidative stress in both chromium toxicity and formation of rhizobial symbiosis and use of nodule bacteria for minimizing toxic effects of chromium on plants.

## 1. Introduction

Heavy metals are widespread environmental pollutants and their excessive levels in agricultural soils cause serious risks not only for normal plant growth and crop yield but also for the human health. Among heavy metals, chromium is a highly toxic metal to living organisms with many adverse effects reported in humans, animals, plants, and microorganisms [1–4]. Chromium belongs to transition metals and it occurs naturally in two predominant valence states: hexavalent chromium (Cr^6+^) and trivalent chromium (Cr^3+^). The hexavalent form of the metal, Cr^6+^, is reported to be more toxic than the relatively less reactive and mobile Cr^3+^ [1]. Hexavalent Cr compounds (mainly chromates and dichromates) are extensively used in diverse fields of industry leading to environmental pollution [1, 5].

Plants, including legumes, are able to uptake heavy metals like chromium from soils that result in many adverse effects, such as inhibition of seed germination and seedling development, reduction in root and shoot biomass, quality of flowers, and crop yield [6–8]. These effects of heavy metals are connected with inhibition of certain metabolic processes, including biosynthesis of chlorophylls and proteins [4, 9–11]. As a result, progressive chlorosis, necrosis, and decreased protein content are typical signs of heavy metal toxicity to plants [1, 12–15].

**Figure 1:**
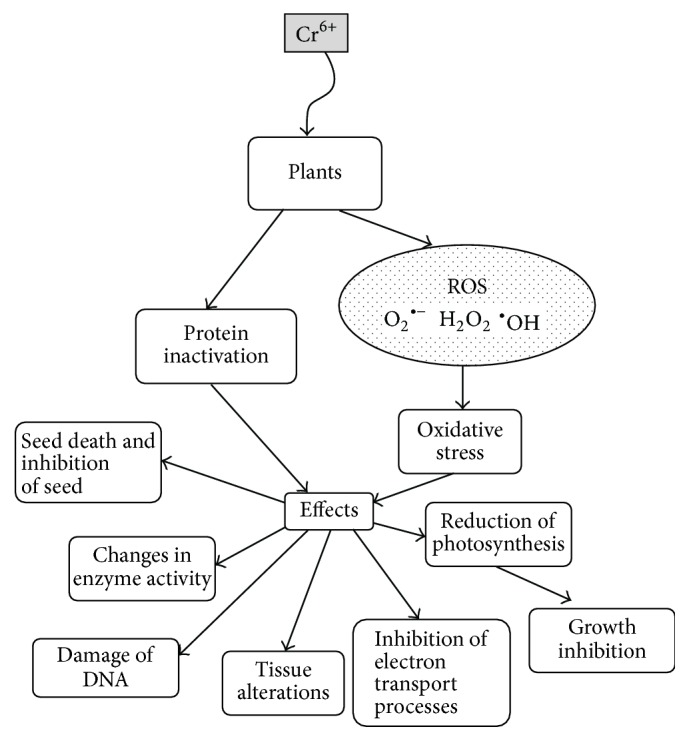
Toxic effects of Cr(IV) on plants. Like other heavy metals, Cr can directly inactivate many proteins binding to them or displacing metals from the active centers of proteins. As a transition metal, Cr can participate in many cellular redox reactions resulting in generation of reactive oxygen species (ROS) such as O_2_^•−^, H_2_O_2_, and HO^•^. When the production of ROS exceeds the capacity of the antioxidant system, cells undergo oxidative stress. Both direct protein inactivation and oxidative stress lead to adverse morphological and physiological changes in plants.

The enhanced production of reactive oxygen species (ROS) is considered to be one of the most important hallmarks of Cr^6+^ toxicity (Figure 1) [3, 5]. ROS, such as superoxide anion radical (O_2_^•−^), hydrogen peroxide (H_2_O_2_), and hydroxyl radical (OH^•^), are highly reactive molecules, which can cause oxidative modification of proteins, lipids, and nucleic acids [3, 12]. In response to heavy metal exposure, plants upregulate various enzymatic or nonenzymatic defense mechanisms that help to support redox balance and prevent/repair oxidative damage under stress conditions (Table 1) [13, 14]. If the capacity of protective systems is not sufficient, modification of biomolecules can be significantly increased, leading to the development of oxidative stress with respective unfavorable effects for plants.

Symbiotic interaction between legume plants and rhizobacteria is a complex physiological process, which is regulated by a number of signals produced both macro- and microsymbionts [16, 17]. Infection by rhizobia increases ROS production, intensifying oxidative processes in plants [17]. There is a strong evidence that ROS and antioxidant system play a key role in the formation and functioning of legume-rhizobium symbiosis [17, 18]. Moreover, a number of studies reported that elevated levels of heavy metals in soils affect rhizobial growth and their host legumes [19, 20]. Under cultivation of legumes on the soils with the high level of heavy metals, the root nodules can be the major accumulators of heavy metals from soil [21]. The latter can potentially reduce the toxicity of heavy metals to the plants with simultaneous decreasing metal content in soils. Therefore, the use of seed inoculation by symbiotic nitrogen-fixing bacteria is actively discussed as one of the possible ways to reduce toxic effects of heavy metals to legumes and provide an effective approach for soil bioremediation [21–23]. At the same time, legume-rhizobium symbiosis seems to be also sensitive to heavy metals, and its protective effects against metal toxicity are not fully clear. This review summarizes recent data on the toxicity of chromium(VI) to plants, especially in legumes with the focus on ROS involvement and oxidative stress development. Furthermore, we analyze ROS role in the formation of rhizobium-legume symbiosis with a focus on modulating effects of nitrogen-fixing bacteria of the* Rhizobium* genus on free radical processes in plants exposed to hexavalent chromium.

## 2. Toxic Effects of Chromium in Plants

In the nature, chromium exists in two valence forms: Cr^3+^ and Cr^6+^, which chromite minerals are mainly composed of [5, 7, 12, 24, 25]. Chromates CrO_4_^2−^ and dichromates Cr_2_O_7_^2−^ are the most abundant anionic forms of chromium in the environment [26–28]. Toxicity of chromium for plants depends on its valence state with Cr^6+^ being more toxic and mobile than Cr^3+^ [12, 29–32]. The hexavalent chromium is toxic for agricultural plants at concentrations of about 0.5–5.0 mg mL^−1^ in the nutrient solution and 5–100 mg g^−1^ in the soil. Under physiological conditions, concentration of chromium ions in plants is less than 1 *μ*g g^−1^ [33, 34].

Absorption of chromium by the plant depends on its form and concentration in soil or water around, as well as plant species and their physiological state [1, 35]. However, the mechanisms of absorption and distribution of chromium in vegetative and generative plant organs have not been sufficiently studied. Chromium is not known as an essential element for plants and, therefore, has no specific mechanisms for assimilation by plants [36]. Both active and passive transport systems were suggested to participate in absorption of this element in plants. Active transport is proposed to be responsible for absorption of chromium ions at low concentrations, whereas passive facilitated diffusion promotes chromium intake at its high concentrations [36]. It is well established that Cr^6+^ is absorbed by roots mainly via an energy-dependent transport, while Cr^3+^ penetrates the plant cell by facilitated diffusion [8, 29, 33, 37]. The absorption of chromium ions by roots is facilitated by organic acids, which are present in root excretions and are able to form complexes with chromium. The latter makes Cr available for absorption by the root system [36]. After absorption by root hairs, chromium is poorly transported to other parts of the plant and is stored mainly in roots [33]. Plant roots accumulate 10–100 times more Cr than shoots and other organs [35, 38]. In particular, growth of* Pisum sativum* L. in the medium containing potassium dichromate caused a dose-dependent increase in Cr content in different plant parts in the following order: roots > stem > leaves > seed [33, 39].

Plants possess certain mechanisms of chromium detoxification [25]. Many plants perform reduction of Cr^6+^ to Cr^3+^ in the thin lateral roots with further transport of Cr^3+^ to the leaves [40]. Soy and garlic plants are known to use this strategy [37, 41]. These plants can reduce Cr^6+^ to the intermediate forms of Cr^5+^ and Cr^4+^, which further are converted to less toxic Cr^3+^ [37, 41]. Plants also protect themselves against Cr toxicity by the immobilization of Cr ions by the cell wall of roots and isolation of Cr in vacuoles [40, 42]. In particular, Cr^3+^ can form highly stable complexes with organic compounds such as peptides (glutathione), carbohydrates (especially pentoses), NADH, FADH_2_, and possibly also organic acids, and these complexes are stored in vacuoles of root cells [37, 43]. Immobilization of heavy metal ions in vacuoles helps to remove them from the metabolically active cell compartments [44].

Accumulation of chromium affects metabolic processes in plants which results in different morphological and physiological defects [12, 29, 30, 45, 46]. Seed germination is primary physiological process, which is influenced by toxic metals. The treatment with 200 *μ*M Cr^6+^ was shown to reduce by 25% germination of* Echinochloa colona* L. seeds [47]. The presence of Cr^6+^ at high concentrations in the soil reduced to 48% seed germination in* Phaseolus vulgaris* L. [48]. Decreased seed germination with increasing concentration of chromium ions was also observed for cowpea* Vigna sinensis* L. [45], melon (*Cucumis melo* L.) [49], and wheat (*Triticum aestivum* L.) [50]. The seed death and delayed seed germination may be explained by activation of proteases or inhibition of amylase activity by chromium with the subsequent decreased transport of carbohydrates to the germ [8, 29].

Among other toxic effects of Cr, the inhibition of root growth was widely observed. Inhibitory effects of chromium on the root elongation were found in wheat and vigna [42, 51]. Another study showed that the exposure to Cr^6+^ in the form of potassium dichromate slightly affected pea germination but inhibited growth of embryonic root and stem of pea plants [52]. The root growth defects under exposure to high levels of heavy metals can be caused by inhibition of root cell division and/or reduction of cell proliferation in the root zone of growth [27, 29, 36, 53]. In general, the toxic effects of heavy metals on roots include (1) reduction of length, biomass, and diameter, (2) damage of the growth cone, (3) destruction of root hairs or decrease in root numbers, (4) increase or decrease of lateral roots formation, (5) increase in lignification, and (6) changes in the structure of the hypodermis and endoderm [8, 29].

The toxicity of heavy metal ions is also manifested in the inhibition of growth of the aboveground parts of plants, reduction of the size of flowers and fruits [6–8]. The reduced plant height because of exposure to Cr^6+^ was described for* Cucumis sativus* L.,* Lactuca sativa* L., and* Panicum miliaceum* L. [54]. Besides that, potassium dichromate reduced the length and mass of roots and shoots of wheat [50] and pea plants [55] in a concentration- and time-dependent manner. Unlike shoots, the root system of pea plants was more sensitive to potassium dichromate exposure that seems to be related to metal accumulation in roots [55].

It has been noted that chromium also affects growth of leaves, the main photosynthetic plant organ. Increasing chromium concentration leads to a significant reduction in the leaf area and leaf biomass, which is accompanied by decreased photosynthesis and induction of chlorosis and necrosis of leaves [6, 8, 56]. Under Cr exposure, many destructive processes take place in leaves. Those include suppression of chlorophyll synthesis, disruption of chloroplast ultrastructure, inhibition of photosynthetic electron transport, and release of magnesium ions from the molecule of chlorophyll [12, 29, 30, 45]. Like other heavy metals, Cr ions can decrease level of carotenoids in some plants [12]. However, we have previously found that cultivation of* P. sativum* with potassium dichromate at different concentrations resulted in higher concentrations of carotenoids and anthocyanins in plant leaves [55]. Assuming that carotenoids and anthocyanins are powerful low-molecular mass plant antioxidants (Table 1), their increased levels may be a result of an adaptive response of pea plants to Cr^6+^-induced oxidative stress of moderate intensity [3, 55].

Hexavalent chromium was found to inhibit CO_2_ absorption and photosynthesis [57], leading to plant biomass reduction [58]. In pea plants, Cr^6+^ added in the form of potassium dichromate significantly decreased photosynthesis, respiration, and symbiotic nitrogen fixation [59]. In spinach, the reduction of photosynthesis induced by Cr^6+^ was connected with inhibition of the electron transport in photosystems I and II within chloroplasts [59]. Cr-induced inhibition of plant growth was also connected with changes in nitrogen metabolism. In particular, chromium treatment adversely affected nitrogenase, nitrate reductase, nitrite reductase, glutamine synthetase, and glutamate dehydrogenase in various plant organs at different growth stages in cluster bean plants [4].

Heavy metals are able to inactivate directly many enzymes via replacement of the primary metal in enzyme active center or causing protein denaturation. Chromium inhibits such enzymes as nitrate reductase [60, 61] and Fe^3+^-reductase in plant roots [62]. In plant mitochondria Cr^6+^ can inhibit electron transport by replacing copper and iron ions in prosthetic groups of many carrier proteins [8, 58, 63]. In fact, Cr^6+^ at concentrations of 20 and 200 *μ*M inhibited cytochrome oxidase in mitochondrial respiratory chain in pea plants [63].

Chromium belongs to transition metals, which participate in cellular redox processes, in particular in ROS production [12, 41, 64]. The latter are intermediates of partial reduction of molecular oxygen and include free radicals such as superoxide anion (O_2_^•−^) and hydroxyl radical (HO^•^) as well as nonradical reactive species, such as hydrogen peroxide (H_2_O_2_) and other peroxides [3]. Being an element with changeable valence, chromium can enter Haber-Weiss/Fenton-type reactions resulting in generation of HO^•^ radical. In the cell Cr^6+^ is reduced by cellular reductants, such as glutathione with an assistance of glutathione reductase, to Cr^5+^, which can further react with H_2_O_2_ in Fenton reaction with HO^•^ formation [3, 41, 56].

(1) Cr6+→thiolsGSH,enzymesCr5+complex→H2O2HO•

(2) Cr6++O2•−⟶Cr5++O2

(3) Cr5++H2O2⟶Cr6++HO•+OH−

Moreover, chromium-induced inactivation electron transport in pea root mitochondria was accompanied by enhanced O_2_^•−^ generation [63]. It is well established that ROS can interact with virtually all cellular components, namely, lipids, carbohydrates, proteins, nucleic acids, and so on. Enhanced ROS production promotes development of oxidative stress, when oxidants damage biomolecules prevailing capacity of defensive mechanisms [3]. In support of it, treatment with chromium compounds was found to intensify in a dose-dependent manner lipid peroxidation in wheat, sorghum, moss, pea, and others [12, 61, 63, 65]. In addition, chromium exposure increased level of carbonylated proteins, which are widely used as a marker of oxidative damage to proteins [66].

To avoid oxidative damage plant cells have evolved complex defense systems including nonenzymatic and enzymatic antioxidants and repair system [67]. Antioxidant system of plants includes (see Table 1) (i) the enzymes that directly scavenge ROS and other free radicals (superoxide dismutase (SOD), catalase, and different peroxidases); (ii) the nonenzymatic antioxidant molecules such as ascorbate, glutathione, *α*-tocopherol, carotenoids, and phenol compounds; (iii) the components of ascorbate-glutathione pathway, which scavenge H_2_O_2_ in a coupled series of reactions by using NAD(P)H; (iv) the enzymes involved in disulfide reduction, thioredoxin and glutaredoxin; (v) metal-binding proteins such as ferritin, phytochelatins, and metallothioneins [15, 68]. Capacity of defense systems in plants is largely modulated by chromium concentration in the nutrient medium. Hexavalent chromium at low concentrations increased the activity of antioxidant enzymes such as superoxide dismutase or catalase in pea plants [12, 69], seedlings of chicken rice [12, 69, 70], vigna [57], and basil plants [30]. At the same time, chromium at high concentrations reduced activities of these enzymes in pea plants [63] and seedlings of chicken rice [69, 70]. Meanwhile, the activation of guaiacol peroxidase was found in different plants treated with high chromium concentrations [19, 30, 71]. One may therefore assume that chromium at low concentrations induces adaptive response in plant tissues that allows plant to tolerate this metal without substantial negative effects. However, when chromium is available at high concentrations, the plant defense systems are not able to cope fully with toxic effects of chromium and even antioxidant enzymes may be damaged.

## 3. Legume-*Rhizobium* Symbiosis

Nitrogen (N) is an essential element for plant growth and development. It is a major component of chlorophylls, amino acids, nucleotides, nucleic acids, coenzymes, vitamins, amines, and other plant constituents [72–75]. To be provided with nitrogen for their needs, plants absorb N in the forms of nitrate (NO_3_^−^) and ammonium (NH_4_^+^) from soil but cannot use free nitrogen gas (N_2_) from the atmosphere. The conversion of atmospheric nitrogen into ammonia is provided by many chemical processes, and biological nitrogen fixation is the most important among them [73, 75].

Biological nitrogen fixation is carried out with a large group of soil free-living, associative, or symbiotic prokaryotes called collectively diazotrophs [72, 74, 76]. They include the nodule bacteria from families Rhizobiaceae, Phyllobacteriaceae, Bradyrhizobiaceae, and Hyphomicrobiaceae. These bacteria are able to establish symbiosis with plants from Fabaceae family and accumulate atmospheric nitrogen to cover over 60% of plant needs in nitrogen [73, 74]. The formation and functioning of legume-rhizobial symbiosis is a complex process with a few coordinated stages, which are regulated by signals from both nodule bacteria and the host plant. The stages include (i) preinfection, (ii) infection with nodule formation, and (iii) functioning of the mature nodule and its death [74, 76].

The symbiotic interaction is initiated, when nodule bacteria infect root hair of the host plant. Chemotaxis of soil nodule bacteria to root exudates plays an important role in the initiation of symbiosis [74, 77]. Both rhizobia and host plants exhibit a strong specificity. To identify rhizobia as benefit partners, the host plants secrete specific compounds, which are recognized by the homologous (compatible) bacteria. The main compounds of the root exudates are carbohydrates, organic acids, amino acids, and phenols [78]. Root exudates attract and induce attachment of compatible rhizobia to walls of the root hair cells [79]. Subsequently, rhizobia adhere to and colonize the root surface. Rhizobia are attached to root hairs by the specific surface polysaccharides, which interact with lectin receptors on root hair cell walls [74, 79, 80]. After attachment, the bacteria interact with certain flavonoids, which are produced by root legumes. Plant flavonoids trigger the expression of bacterial nodulation genes (*nod*-genes), which control the formation of nodules in plant roots [79, 81, 82]. The products of* nod*-genes are involved in synthesis and export of specific lipochitooligosaccharides called Nod factors. Bacterial Nod factors serve as signaling molecules that initiate nodule formation in root cortex [83, 84]. Previously it has been assumed that binding plant lectins to bacterial surface polysaccharides plays a key role in the specificity between rhizobia and their legume hosts. In accordance with recent data the host specificity of legume plants is presumably determined by the Nod factors and to a lesser extent by surface polysaccharides of the nodule bacteria [85]. In addition to Nod factors, rhizobial surface polysaccharides, such as exopolysaccharides, lipopolysaccharides, capsular polysaccharides, and cyclic glucans, are also important for development of root nodules and modulation of host specificity. In particular, binding of plant lectins to bacterial polysaccharides can influence adhesion of bacteria to root hairs in pea plants: the mutant* R. leguminosarum* strain, defective in synthesis of surface glucomannan, had an impaired ability to attach to root hairs [86]. Recently, exopolysaccharide receptor has been identified in* Lotus japonicus* that controls rhizobial infection and distinguishes between compatible and incompatible exopolysaccharides [87].

In general, the formation of a nodule requires the reprogramming of differentiated root cells to form a primordium, which a nodule can develop from. The bacteria enter the developing nodule via formation of infection threads. Regulation and stages of root nodule formation have been comprehensively reviewed previously [15, 88–91]. The nodule formation is completed when nodule bacteria are transformed in nitrogen-fixing bacteroides [92, 93]. The formed nodules may be either determinate or indeterminate depending on the host. Determinate nodules have a short-lived meristem, and they grow by plant cell expansion and division, resulting in nodules progressing through well-defined developmental stages. Legumes, which formed determinate nodules, include* Lotus *sp.,* Phaseolus* sp., and* Glycine max*. In contrast, indeterminate nodules have a persistent meristem and infection is continuous. New nodule cells are subsequently infected by rhizobia residing in the nodule.* Medicago *sp.,* Vicia *sp.,* Trifolium* sp.,* and P. sativum* are typical legumes with indeterminate nodules [94].

In nodules bacteroides are provided with microaerobic environment required for expression of enzymes of the nitrogenase complex. A plant-produced oxygen-binding protein, called leghemoglobin [15], controls oxygen supply to bacteroides. Nitrogenase complex located on internal membranes of bacteroides is responsible for ATP-dependent reduction of free nitrogen to ammonia [79, 95]. Further, ammonia interacts with intracellular keto acids (*α*-ketoglutaric, pyruvic, or oxalic acids) in dehydrogenase- and transaminase-catalyzed reactions forming respective amino acids, such as glutamine, alanine, or asparagine [96]. In the form of free ammonia, amino acids or amides, nitrogen-containing substances are transported from nodules to the roots, and then to the aboveground parts of plants [97].

Biological nitrogen fixation is closely connected with photosynthesis, since the latter provides assimilates and energy resources to nodule bacteria, and the bacteria, in turn, provide photosynthetic apparatus of plants with nitrogen compounds [96, 98]. The intensity of photosynthesis and ammonium inclusion in the plant metabolism depends on content and functional activity of chloroplasts, the structural elements of the photosynthetic apparatus [99]. At the same time, products of bacterial nitrogen fixation substantially affect the intensity of photosynthesis and transport of photoassimilates from plants to nodules [100]. Thus, this is a real symbiosis providing mutual benefits for both partners, plants and bacteria.

## 4. Free Radical Processes in Legume-Rhizobium Symbiosis 

To date, there is much evidence that ROS and antioxidant defense play an important role in the formation and functioning of legume-rhizobium symbiosis [101, 102]. Similarly to pathogen invasion response, the infection of legumes with rhizobia causes an intensification of oxidative processes in plant cells, promoted by increased production of ROS and nitric oxide (NO^•^). However, apart from response to pathogenesis, production of ROS and NO^•^ may not be a plant defense response to the rhizobia but rather a process that is needed for the development of a symbiosis. Elevated levels of ROS were found to be necessary for the effective penetration of bacteria into plant tissues, since the decrease of ROS and NO^•^ levels prevented formation of bacterial infection thread and delayed nodule formation [101–103].

Like jasmonic acid and ethylene, ROS, particularly O_2_^•−^ and H_2_O_2_, were supposed to act as signaling molecules, regulating formation of senescence of legume-rhizobium symbiosis [104–106]. At the initial stages of symbiosis, an oxidative burst occurs in the place of bacterial infection [107]. Recent studies suggest that legume NADPH-oxidases play a pivotal role in ROS production under oxidative burst and, in turn, have a crucial role in different stages of nodulation [108]. Oxidative burst can have a dual function in legume-rhizobium symbiosis: it inhibits the protective reactions of plants on penetration of compatible bacteria, or, conversely, it can activate the protective mechanisms of plants under adverse conditions for symbiosis [109, 110]. Accordingly, bacterial Nod factors were shown to stimulate oxidative burst by blocking the induction of* nod*-genes in plants when the interaction between symbionts is incompatible [110, 111].

The free radical processes in plant cells largely depend on many exogenous and endogenous factors [17]. In particular, alkalization of the cytoplasmic pH causes membrane depolarization and increased the interaction of plant cells with rhizobia [76]. Plant phenolic compounds, which are susceptible to rhizobial infection, can undergo autoxidation by free oxygen and thereby increase ROS levels, in particular H_2_O_2_ [17]. Production of H_2_O_2_ during symbiosis was detected in infection threads and root nodules of* Medicago sativa* and* P. sativum* [107, 112]. Hydrogen peroxide is relatively long-living ROS and can easily diffuse via biological membranes and act at distant places. Dependently on the concentration H_2_O_2_ can directly act as an antibacterial agent or as a signal molecule triggering adaptive response in plants [17, 101, 113]. In addition, H_2_O_2_ has been shown to be necessary for the optimal propagation of infectious bacterial threads inside root hairs and membranes of plant cells [114].

Production of ROS in legume-rhizobium symbiosis also occurs during the reductive processes required for nitrogen fixation. Many compounds that act as electron donors for nitrogenase (e.g., ferredoxin) are able to autoxidize with O_2_^•−^ [83]. ROS production may be also promoted by leghemoglobin, which is present in nodules at high levels. In the presence of O_2_, leghemoglobin can undergo autoxidation and, as a result, O_2_^•−^ is generated with further dismutation to H_2_O_2_ [83]. The interaction of leghemoglobin with H_2_O_2_ leads to the formation of a highly oxidized ferric-porphyrin cation-radical, which further can oxidize protein molecules with formation of, for example, tyrosine radicals [83, 115]. In addition, H_2_O_2_ can be released from leghemoglobin and promote HO^•^ generation via Fenton reaction [115].

Nitrogenase complex in bacteroides is very sensitive to ROS; therefore, it not surprising that legume nodules have efficient mechanisms to maintain proper redox balance and low ROS levels. Like plants, nodules possess a powerful system of antioxidant defense consisting of antioxidant enzymes (SODs, catalase, and various peroxidases), enzymes of the ascorbate-glutathione cycle, and low-molecular mass antioxidant metabolites such as ascorbate, glutathione, and tocopherols (Table 1) [68, 116, 117]. The capacity of nodule antioxidant system affects largely nitrogen-fixing efficiency; in particular, nodules may not function without ascorbate-glutathione cycle [68].

Previous studies have shown that changes in O_2_^•−^ and H_2_O_2_ levels in* P. sativum* roots under symbiosis development with nodule bacteria depend on the efficacy of rhizobial strains and the ability of peas to form nodules [17]. Significantly increased levels of O_2_^•−^ and H_2_O_2_ were found in the pea roots after inoculation by incompatible strains of bacteria* Rhizobium leguminosarum* bv.* phaseoli*. This may indicate ROS involvement in protection against infection of the pea roots with rhizobia [17]. Differential changes in the activities of SOD, catalase, and peroxidase were found in pea roots inoculated by different rhizobial strains [17, 101]. In response to inoculation with highly effective* Rhizobium* strain, the activity of the antioxidant enzymes in pea plants did not increase and coincided with the decrease in ROS levels in the plants roots. Such a correlation between ROS levels and the antioxidant system activity was supposed to determine interaction of bacteria and plants to promote their effective symbiosis. At the same time, enhanced SOD and peroxidase activities in pea plants after inoculation by incompatible strain* R. leguminosarum* bv.* phaseoli* could result from increased O_2_^•−^ generation. The increased antioxidant enzyme activity can prevent an increase in the level of O_2_^•−^ to a critical value but complicate the development of symbiosis [17].

The inoculation of pea roots by effective* R. leguminosarum* bv.* viciae* strains increased O_2_^•−^ and H_2_O_2_ levels with simultaneous stimulation of antioxidant enzymes in pea seedling epicotyls. However, ROS are not directly involved in the development of infection and subsequent formation of nodules [118]. This suggests that the plants have certain mechanisms to prevent bacterial infection in organs that cannot form nodules [17]. It is supposed that limitation of rhizobial infection is connected with triggering a reaction similar to the systemic acquired resistance in phytopathogenesis [101] or systemic induced resistance as in the case of infection by nonpathogenic microorganisms [17, 119]. It is known that ROS can upregulate expression of genes encoding hydrolytic enzymes, stress-protective proteins, enzymes involved in synthesis of phenolic compounds, phytotoxins, and other substances required for development of acquired resistance to pathogens [120].

Thus, ROS generation is among key components of the plant response to infection with both compatible and incompatible bacteria. Plants delicately regulate ROS levels using mechanisms of ROS generation and activation of antioxidant system [17]. Notably, at the later stages of symbiotic formation, when the amount of rhizobia in the roots reaches a certain level, the host plant may also include mechanisms of ROS generation and activation of the antioxidant system to regulate further nodule formation [117]. In addition, during nodule senescence high ROS levels have been detected in senescing symbiosomes suggesting ROS involvement in this process [18].

## 5. Effects of Legume-Rhizobium Symbiosis and Cr(VI) Toxicity

The ability of nodule bacteria to form a symbiosis with legume plants depends on many environment factors, such as temperature, humidity, aeration, pH medium, soil structure, presence of labile nitrogen forms, phosphorus, potassium, and magnesium in the soil [121, 122]. In addition, microorganisms are very sensitive to the presence of heavy metal ions in the soil [123]. A number of heavy metals (e.g., Cu, Ni, Zn, Cd, and As) have been reported to inhibit the growth and modify morphology structure and activities of various groups of soil microorganisms including symbiotic nitrogen fixators like* Mesorhizobium ciceri*,* Rhizobium *sp.,* Bradyrhizobium* sp., and* Sinorhizobium *sp. [124]. Among mentioned metal elements a strong inhibitory effect of copper on growth and enzyme activities of* Bradyrhizobium* BMP1 strain was found [124]. Effective* R. leguminosarum *bv.* trifolii *population did not survive during long-term incubation in soils containing 7.1 mg Cd kg^−1^ [125]. Heavy metals, including Cr, inhibit the activity of nitrogenase in nodules leading to decreased intensity of nitrogen fixation [72]. Mechanisms of chromium toxicity to nodules are not well studied, but one may suggest they include oxidative stress development and protein modification. Since nitrogenase is very sensitive to oxidation, Cr treatment can lead to inactivation of the enzyme and impair functioning of nodules.

Several studies have reported that nitrogen-fixing bacteria can diminish the toxicity of heavy metals on host plants [19–21, 126]. The protective effects are proposed to be associated with the accumulation of the metals in the nodules. Accordingly, the nodule bacteria are exposed more to heavy metals than the host plant. Resistance of the bacteria to heavy metals is both species- and strain-specific [126]. One can suppose that if bacteria have powerful defense mechanisms against heavy metal toxicity, the protective effect of these bacteria on the host plant will be more pronounced.

**Figure 2:**
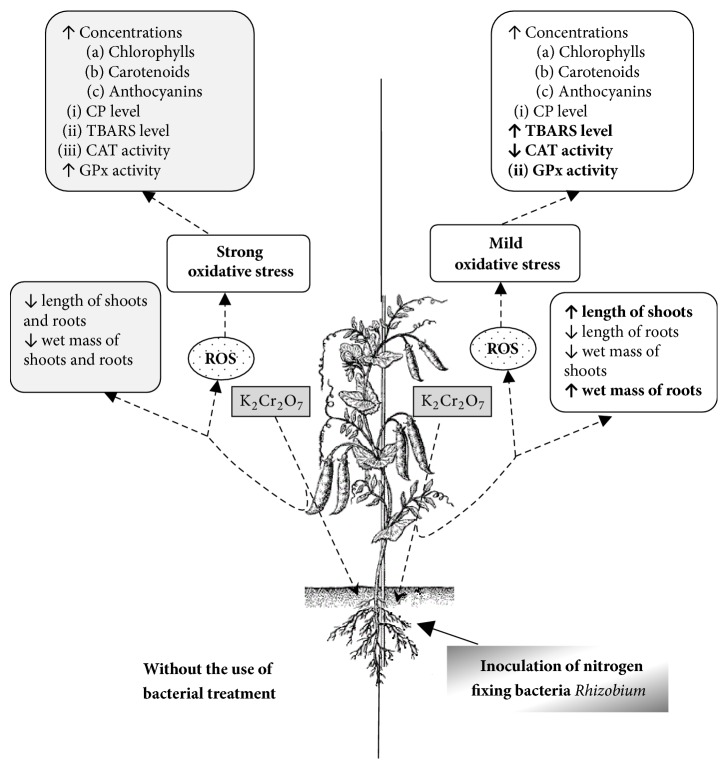
Effects of Cr(IV) exposure alone and in combination with nodule rhizobacteria on selected growth parameters and ROS homeostasis in* P. sativum* plants. Arrows ↑ and ↓ indicate the increase and decrease in the parameter, respectively.

Like other bacteria, protective mechanisms of rhizobia against chromium toxicity apparently include direct and indirect reduction of Cr^6+^ to Cr^3+^, metal binding with further isolation or elimination, and upregulation of antioxidant defense [1]. In the case of exposure to Cu, the rhizobial symbiosis with* Sinorhizobium meliloti* CCNWSX0020 also upregulated expression of genes encoding components of antioxidant defense in both, plants and bacteria. The results indicated that the rhizobial symbiosis with* S. meliloti* CCNWSX0020 not only enhanced plant growth and metal uptake, but also improved the responses of plant antioxidant defense to Cu excess [126]. Regarding Cr influence, we have recently found that inoculation with highly effective nitrogen-fixing bacteria decreased the toxic effects of chromium (IV) on* P. sativum*. The protective effects included improvement of the length of shoots and mass of the plant roots and enhanced levels of chlorophylls, carotenoids, and anthocyanins compared with the effects of chromium on pea plants without inoculation (Figure 2) [127]. In addition, treatment with potassium dichromate did not affect level of oxidized proteins but increased levels of lipid peroxidation products and decreased catalase activity in plants preinoculated with nitrogen-fixing rhizobia, but not in noninoculated pea plants [127]. Despite increased levels of lipid peroxidation products, pea plants grew better when treated with Cr in the presence of nodule bacteria if compared with the plants treated only with Cr. We suppose that nodule bacteria are able to decrease Cr toxicity to pea plants and their protective effects could be connected rather with modulation of synthesis of plant pigments than with involvement of enzymatic antioxidant defense. Since carotenoids and anthocyanins have antioxidant properties, they might be involved in minimization of negative effects from oxidative stress induced by chromium. It is possible that accumulation of chromium in root nodules decreased Cr transport to other plant parts allowing the latter to develop protective mechanisms that are more effective.

## 6. Conclusions and Perspectives

The presence of chromium compounds in soils inhibits seed germination and induces various morphological and physiological defects in many plants, including legumes. Toxicity of chromium in plants is connected with the enhanced ROS formation and oxidative stress development resulting in the intensified protein modification, lipid peroxidation, and DNA damage. In legume-rhizobial symbiosis both host plant and nodule bacteria undergo oxidative stress induced by chromium, with rhizobia being more stressed due to preferential Cr accumulation in root nodules. Available data suggest that inoculation with nodule bacteria can be considered as an effective approach to minimize toxic effects of chromium and other heavy metals on agricultural plants. At the same time, the protective efficacy of nodule bacteria depends on many factors such as type and concentrations of metals, compatibility of partners, virulence, adaptive capacity, and nitrogen-fixing activity of bacteria. Therefore, the effects of heavy metal on legume-rhizobium symbiosis and search of ways to enhance metal resistance of nodule bacteria are perspective potential research direction. It seems that Cr at low concentrations may induce mild oxidative stress [128], which can have beneficial rather than detrimental effects on legume-rhizobium symbiosis and plant metabolic processes (Figure 2). As known, mild oxidative stress may induce adaptive response, which enhances resistance to many lethal stresses [128]. In addition, mild oxidative stress plays an important role in establishment of effective legume-rhizobium symbiosis [103]. We propose that using the pretreatment with nodule bacteria at low levels of oxidants can aid bacteria to resist high levels of heavy metals in the environment. The construction of nodule bacterial strains with higher resistance to environmental stresses may be a great opportunity to increase the benefit from their use in bioremediation and cultivation at polluted areas.

## Figures and Tables

**Table 1:** Major components of antioxidant defense in legume plants and root nodules.

Components	Location	Function
*Enzymatic antioxidants*		
Superoxide dismutase (SOD)		Dismutation of O_2_^•−^: O_2_^•−^ + O_2_^•−^ + 2H^+^ → 2H_2_O_2_ + O_2_
Cu, Zn-SOD	Cytosol, plastids	
Mn-SOD	Mitochondria, bacteroides	
Fe-SOD	Plastids, some bacteroides	
Catalase	Peroxisomes, bacteroides	Dismutation of H_2_O_2_: 2H_2_O_2_ → 2H_2_O + O_2_
Guaiacol peroxidase (GPX)	Plasma membrane, vacuoles	Detoxification of H_2_O_2_: H_2_O_2_ + guaiacol_red_ → H_2_O + guaiacol_oxid_
Peroxiredoxins	Cytosol, mitochondria	Reduction of H_2_O_2_ or alkyl hydroperoxides (ROOH) to H_2_O or respective alcohols (ROH): ROOH + Trx-(SH)_2_ → ROH → Trx-S_2_ + H_2_O
*Enzymes of ascorbate-glutathione cycle (AA-GSH cycle)*		
	Cytosol (mainly), mitochondria, plastids, peroxisomes	Detoxification of H_2_O_2_ in a series of reactions
Ascorbate peroxidase (APX)		*APX:* H_2_O_2_ + AA → 2H_2_O + MDHA (DHA)
Monodehydroascorbate reductase (MR)		*MR:* 2MDHA + NADH → 2AA + NAD
Dehydroascorbate reductase (DR)		*DR:* DHA + 2GSH → AA + GSSG
Glutathione reductase (GR)		*GR:* GSSG + NADPH → 2GSH + NADP^+^
*Enzymes of GSH metabolism*		
Glutathione peroxidase (GSH-PX)	Cytosol, plastids, bacteroides	Detoxification of H_2_O_2_: H_2_O_2_ + GSH → 2H_2_O + GSSG
Glutathione reductase (GR)	Cytosol, mitochondria, plastids, bacteroides	Regeneration of oxidized glutathione: GSSG + NADPH → 2GSH + NADP^+^
*α*-Glutamylcysteine synthetase and glutathione synthetase	Cytosol, plastids, bacteroides	GSH synthesis de novo
Glutathione-S-transferase	Cytosol (mainly), plastids mitochondria, bacteroides	Detoxification of xenobiotics via conjugation with GSH

*Nonenzymatic antioxidants*		
Ascorbic acid (AA)	Cytosol, mitochondria, plastids, peroxisomes	Direct scavenging of ROS, a substrate for APX
Glutathione (GSH)	Cytosol, mitochondria, plastids, bacteroides	Direct scavenging of ROS, a cosubstrate for DR, GSH-PX, and glutathione-S-transferases
*α*-Tocopherol	Plastids	Direct scavenging of ROS, quenching of lipid radicals in membranes
Carotenoids	Plastids	Quenching of electron-excited molecules, protection of chlorophylls from photodamage
Phenol compounds (phenolic acids, flavonoids, etc.)	Vacuoles (mainly), cytosol, plastids, cell wall	Direct scavenging of ROS and lipid radicals, metal-chelating activity
Ferritin, phytochelatins, and metallothioneins	Plastids, vacuoles, cytosol	Metal-binding activity

Trx-(SH)_2_/Trx-S_2_: reduced/oxidized thioredoxin; MDHA: monodehydroascorbate; DHA: dehydroascorbate; GSH/GSSG: reduced/oxidized glutathione.
